# Glasgow Royal Infirmary

**Published:** 1833-03

**Authors:** 


### GLASGOW ROYAL INFIRMARY.

*Compound Fracture of the Right Tibia; Secondary Hemorrhage, and diffuse Inflammation of the Cellular Texture; Death.*

W.P., admitted 27th June, 1826, on account of a compound fracture of the right leg, three inches above the ankle. The injury was occasioned by the falling of a quantity of building materials on the limb. The integuments on front of the leg were lacerated to the extent of two inches, through which an oblique portion of the upper shaft of the tibia protruded; the fibula was also fractured, as were one or two of the metatarsal bones. The pulsation of the anterior and posterior tibial arteries was readily distinguished at the ankle; the limb was therefore put up in the usual manner, and laid on its outer side. A few hours after his admission, smart febrile excitement took place, and continued for several days, requiring bloodletting, purgatives, diaphoretics, and opiates. The dorsum of the foot was swollen and painful, and, on removing the bandage, it was found red and vesicated; these were speedily removed by leeches and a cold lotion. At the same time he complained of pain over the tibia, a little below the knee, which was subdued by the same treatment. On the eighth day, hemorrhage took place from the wound to the amount of ten ounces, which was checked by the application of a tourniquet. At a consultation immediately after this occurrence, the splints and bandage were removed, and the wound found sloughy, and covered with clotted blood. A splint was applied to the outer, and another to the inner side of the leg, and retained by two strips of bandage, the displacement of the bones being prevented without producing unnecessary pressure. The front of the limb was thus exposed, and the wound covered with a pledget of lint, dipped in camphorated oil. The pulse was one hundred and thirty, feeble; the countenance pale and anxious, and the skin cold; but in half an hour this state of collapse, produced by the loss of blood and the alarm which it excited, was got rid of; the pulse continued as frequent, but became full and jerking; the skin hot and dry, the tongue parched, the thirst urgent, and the breathing accelerated, with a tendency to rigor. It was not thought advisable to disturb the sloughy cake which covered the front of the tibia, to ascertain and secure the vessel from which the bleeding proceeded. The leg was swollen to more than twice its natural size, and had that boggy feel so characteristic of extensive destruction of the cellular tissue. Free incisions were had recourse to, and the affected parts covered with an evaporating lotion. He had other three attacks of hemorrhage, but the quantity of blood lost did not exceed six ounces; the febrile excitement increased, and became typhoid; the strength declined rapidly, and he died on the twelfth day from the receipt of the injury. On inspection, it was ascertained that the hemorrhage had proceeded from the posterior tibial artery opposite the fracture; the wound was sloughy, as was the subcutaneous and inter-muscular cellular tissue through the whole leg. There was slight serous effusion under the arachnoid and into the ventricles of the brain, and a few tubercles in the upper lobe of the left lung.

The fatal termination of this case was to be attributed, not so much to the attacks of hemorrhage, as to the diffuse inflammation of the cellular texture, and the typhoid symptoms with which it was accompanied. Had the bleeding occurred soon after the injury, there could have been no hesitation as to the propriety of immediately securing the wounded vessel; but it did not take place till the eighth day, when the gangrenous state of the wound rendered an attempt of this kind altogether unjustifiable. There still remained, however, two modes of procedure which might have been adopted. The first consisted in tying the artery from which the hemorrhage proceeded, at some distance from the wound; and the second, in amputation of the limb.

When a limb is extensively inflamed, swollen, and puffy, which frequently happens in the course of eight or ten days after the receipt of such injuries, it becomes difficult to secure the artery; the usual land-marks by which we are guided are obscured, a greater extent and depth of incision are required, and that, too, in an unhealthy part. If gangrene be impending, the result of this operation will generally prove unsuccessful. I have seen one case, in which the anterior tibial artery was tied three inches above the fracture, for profuse hemorrhage on the eleventh day, and where gangrene supervened, and the patient died. The comparatively trifling amount of the hemorrhage in the case last detailed, and the fact, that the alarming state of the patient did not depend so much on the loss of blood as on the diffuse inflammation of the leg, and the high constitutional excitement which it produced, were the reasons which induced me to defer securing the bleeding vessel by ligature.

The propriety of amputation was also the subject of consideration. Here the question naturally presented itself, under what circumstances are we justifiable in adopting this violent measure, on account of secondary hemorrage from a compound fracture? Should it be found impracticable, from the sloughy state of the wound, to secure the bleeding mouth of the vessel; or should the inflamed and swollen state of the limb, and the extensive destruction of its cellular tissue, be such as to prevent a ligature from being applied to the affected vessel, at some distance from the injury; or should the hemorrhage recur from the activity of the collateral circulation after this had been performed, we should then be warranted in having recourse to amputation. This was not, however, thought advisable in the last case; because, from the typhoid character of the symptoms, and the alarming state of irritability and exhaustion which existed, we had reason to dread that the shock of the operation, and the loss of blood which must have attended it, would prove speedily destructive.

When the injury is followed by profuse hemorrhage, and the ensuing collapse is so severe and protracted as to prevent amputation from being immediately had recourse to, the subsequent reaction, when it does take place, is often unusually violent, and is apt, if not subdued, or at least greatly moderated, to interfere with the healing of the stump, and end fatally, by the formation of purulent depôts in some of the thoracic or abdominal viscera.—*Macfarlane's Surgical Reports.*

